# Research on key indicators for diagnosis and prediction of rheumatoid arthritis based on GBDT+LR embedded feature selection model

**DOI:** 10.3389/fimmu.2025.1679223

**Published:** 2026-01-09

**Authors:** Jiaqi Chen, Qiang Zhang, Zhenqiang Huang, Chunsheng Qu

**Affiliations:** Clinical Laboratory of The Lishui Hospital of Wenzhou Medical University, The First Affiliated Hospital of Lishui University, Lishui People's Hospital, Lishui, China

**Keywords:** diagnostic model, embedded feature selection, GBDT+LR Model, rheumatoid arthritis, SHAP (SHapley Additive exPlanations)

## Abstract

**Background:**

Rheumatoid arthritis (RA) exhibits substantial diagnostic overlap with other autoimmune diseases that share similar pathological features, leading to redundant testing and limited diagnostic specificity. Therefore, there is an urgent need to identify critical clinical indicators with high diagnostic and predictive value to improve both diagnostic efficiency and accuracy.

**Methods:**

To address this challenge, we propose a multidimensional embedded feature selection framework based on ensemble learning. This framework integrates Gradient Boosted Decision Trees (GBDT) and Logistic Regression (LR) models to extract potential diagnostic features from multi-source clinical datasets. GBDT captures complex nonlinear interactions among features, enhancing adaptability to heterogeneous data, while LR leverages its sparsity-promoting characteristics to perform dimensionality reduction and highlight discriminative variables. To further improve interpretability, the SHapley Additive exPlanations (SHAP) algorithm was employed to quantify the contribution of each feature to the model’s predictions and to identify novel diagnostic markers beyond traditional indicators.

**Results:**

Validated on real-world clinical data, the proposed framework achieved excellent diagnostic performance across multiple evaluation metrics, significantly enhancing the specificity and accuracy of RA diagnosis. Compared with conventional diagnostic methods, our model demonstrated marked improvements in test accuracy and area under the receiver operating characteristic curve (AUC). SHAP not only reaffirmed the importance of RF and anti-CCP but revealed that systemic metabolic indicators—such as low HDL, elevated bile acids, and altered creatinine—carry independent diagnostic weight. This supports a paradigm shift toward viewing RA as a multi-system inflammatory disorder, enabling earlier clinical suspicion even before classic articular manifestations.

**Conclusion:**

The proposed multidimensional embedded feature selection framework showed strong diagnostic performance and interpretability in identifying key biomarkers for RA, effectively addressing the issue of indicator redundancy and enhancing diagnostic precision. This pragmatic application of an established GBDT+LR framework, integrated with SHAP for interpretability and built on routine clinical data, offers potential clinical utility in RA diagnosis.

## 1 Introduction

Rheumatoid arthritis (RA) is a chronic autoimmune disease characterized by synovial inflammation and bone erosion, which can ultimately lead to joint destruction and functional disability (1). Its pathogenesis is multifactorial, involving genetic predisposition, immune dysregulation, and environmental triggers, with the production of autoantibodies such as rheumatoid factor (RF) and anti-citrullinated protein antibodies (ACPAs) recognized as central immunological features (2). The global prevalence of RA in adults is estimated at 0.5% to 1%, with a markedly higher incidence in females, typically manifesting in midlife. The disease imposes a substantial burden on public health due to its chronicity and progressive disability potential (3, 4).

A major challenge in the clinical diagnosis of RA lies in its substantial overlap with other autoimmune diseases that share similar immunopathological features. Although RF exhibits moderate specificity for RA, it can also be detected in other autoimmune and infectious diseases. Likewise, inflammatory markers such as C-reactive protein (CRP) lack disease specificity due to their broad-spectrum nature (5). As a result, current diagnostic practice often relies on broad screening strategies rather than targeted assays, leading to increased healthcare costs and limiting the timely and accurate identification of RA cases (6). While public RA datasets exist, few concurrently include rheumatoid arthritis (RA), its clinical variants (RhA), other autoimmune conditions, and healthy controls with comprehensive, routinely available laboratory panels. Our dataset was specifically curated to address the real-world challenge of differential diagnosis using only standard clinical tests, enhancing deployability in general practice.

In clinical settings, RA must be distinguished from other autoimmune conditions such as rheumatoid arthritis variants (RhA) and systemic lupus erythematosus (SLE), which may present with overlapping serological and clinical features. Commonly used serological biomarkers include anti-cyclic citrullinated peptide antibodies (anti-CCP), RF, and CRP. Anti-CCP exhibits high specificity (up to 95%), while RF is more sensitive but less specific, frequently yielding false positives in non-RA autoimmune diseases. CRP, although dynamically responsive, primarily reflects systemic inflammation and lacks diagnostic specificity. In the early stages of RA, these traditional markers show reduced sensitivity—anti-CCP is positive in fewer than 60% of early-stage patients, and RF can have a false-positive rate of up to 20% (2–4). These limitations contribute to a significant risk of misdiagnosis or underdiagnosis in early RA.

Emerging biomarker studies have revealed that RA patients often exhibit elevated levels of pro-inflammatory cytokines in peripheral blood, and altered glycosylation patterns of immunoglobulin G (IgG) are negatively correlated with disease activity (4). Metabolomic analyses have further shown downregulation of metabolites related to mitochondrial function in RA patients (5). These findings offer a biological rationale for mining high-dimensional clinical and molecular data to improve diagnostic precision. However, traditional manual or univariate analytic approaches fall short in handling the complexity and interaction effects within such data (6).

In recent years, machine learning (ML) techniques have demonstrated distinct advantages in analyzing unstructured and high-dimensional heterogeneous data across various domains (7–9). Ensemble learning methods, in particular, have shown great promise in medical diagnostics due to their robustness and adaptability (10–12). In bioinformatics, multi-omics diagnostic models for RA have received increasing attention. Researchers have applied ML algorithms—such as random forests, support vector machines, and neural networks—for feature selection and pattern recognition. Nonetheless, existing models often suffer from key limitations: (1) reliance on single-omics or homogeneous data types, reducing their generalizability across datasets (13, 14); (2) lack of interpretability, hindering biological validation (15, 16); and (3) insufficient modeling of nonlinear and higher-order feature interactions, which may compromise diagnostic performance (16, 17).

To address these limitations, we constructed a multiclass clinical dataset comprising RA, RhA, and healthy controls, incorporating a broad range of routinely collected laboratory indicators. We propose an embedded feature selection framework that integrates Gradient Boosted Decision Trees (GBDT) and Logistic Regression (LR) to effectively capture nonlinear feature interactions while reducing dimensionality through sparsity-driven modeling. Furthermore, we employ the SHapley Additive exPlanations (SHAP) framework to interpret the contribution of individual features to model predictions, aiming to identify diagnostically meaningful combinations of indicators that extend beyond conventional biomarkers.

## 2 Methods

**Figure 1:**
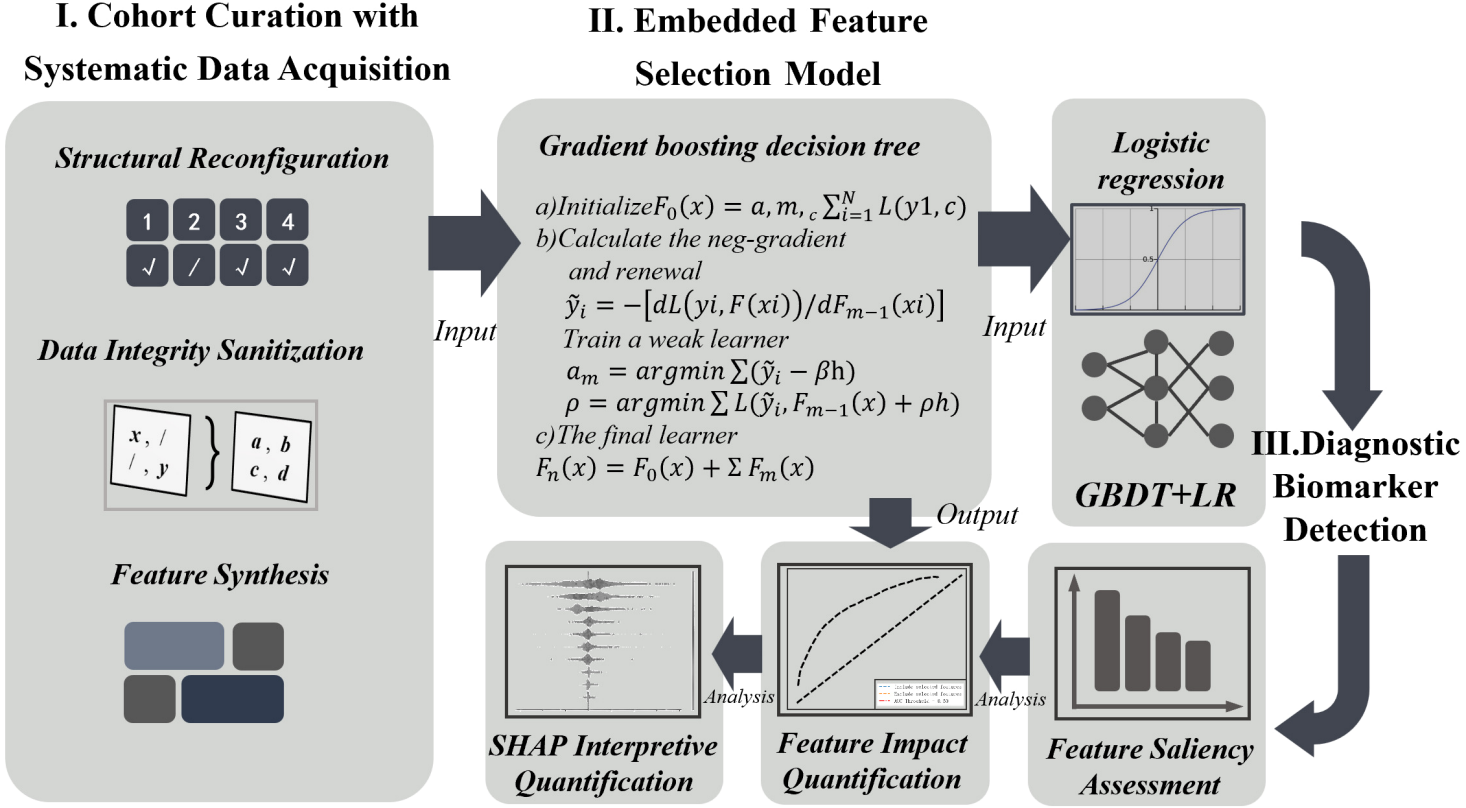
Flowchart of the study design.

The overall research workflow is illustrated in **Figure 1**.

### 2.1 Data sources and standardized characteristics construction

This study included 842 cases comprising patients with rheumatism-related conditions who visited the Department of Rheumatology and Immunology at Lishui People’s Hospital from January to December 2023, as well as healthy individuals undergoing routine physical examinations during the same period. The inclusion criteria were: (1) age ≥18 years; (2) availability of complete clinical laboratory data; and (3) a definitive diagnosis of rheumatoid arthritis (RA), rheumatoid arthritis variant (RhA), other autoimmune diseases, or no history of rheumatic disease as determined by a rheumatologist. Exclusion criteria were: (1) presence of acute infections, malignancies, or other severe comorbidities; (2) recent use of corticosteroids or immunosuppressants that could influence immune function; and (3) significant missing data or evident data anomalies.

All laboratory indicators were obtained from routine diagnostic tests performed during clinical visits. These included:

1. Biochemical indicators: liver and kidney function, electrolytes, blood glucose, blood lipids, etc.;
2. Immunological markers: RF, anti-CCP, antinuclear antibody (ANA) spectrum, immunoglobulins, complements, etc.;
3. Hematological parameters: leukocyte count, red blood cells, platelets, and differential cell counts.

After data collection, all samples underwent standardized preprocessing procedures including missing value imputation, outlier detection and correction, and variable normalization.

Based on multi-source clinical data exported from the hospital information system (HIS)—encompassing eight fields such as demographic information, rheumatologic tests, and complete blood counts—standardized data processing was implemented in three stages:

Step 1: Structural reconstruction

Raw unstructured data were converted into a relational database format, using “patient name + visit ID” as the unique index. Test results were column-aligned, and irrelevant fields were eliminated.

Step 2: Data cleaning and integrity enhancement

Twenty-three clinically relevant indicators (e.g., RF, CRP) were retained according to rheumatology diagnostic guidelines. Records with >15% missing key variables were excluded (n = 47). For non-key missing values, k-nearest neighbor (KNN, k = 5) imputation was applied. Outliers were corrected using Tukey’s fences method (interquartile range = 1.5), resulting in a 7.3% correction rate.

Step 3: Knowledge-driven feature engineering

Based on known RA pathophysiology, features were grouped into thematic “buckets” (e.g., inflammatory response, autoantibodies) (18). Derived features were constructed, such as the CRP/IL-6 normalized ratio and RF × IgG interaction term. The final dataset consisted of a high-dimensional feature matrix integrating both original and engineered indicators.

### 2.2 Preliminary feature selection using statistical methods

Given the unknown sampling mechanism and distributional properties of the collected clinical data, and the possibility of violating normality assumptions, initial statistical screening was performed to support subsequent embedded feature analysis.

To evaluate inter-group variability in feature distributions across diagnostic categories (RA, RhA, other autoimmune diseases, and healthy controls), one-way analysis of variance (ANOVA) or Kruskal–Wallis H tests were used depending on distributional characteristics. Features with statistically significant variation among groups were retained as candidates for downstream modeling.

To enhance robustness, a hierarchical statistical feature selection framework was established, consisting of:

Distributional testing: The Shapiro–Wilk test was applied to evaluate normality for each feature, distinguishing between normally and non-normally distributed variables.

Variance testing: ANOVA was used for normally distributed features; Kruskal–Wallis H tests were applied to non-normal features to determine distributional differences across disease categories.

Multiple hypothesis correction: The Bonferroni method was used to control Type I error rates, with an adjusted significance threshold set at α = 0.01.

Effect size evaluation: Features with effect size > 0.5 were retained, as determined by Cohen’s d (for continuous variables) or Cramér’s V (for categorical variables).

The initial dataset included 170 routinely collected clinical variables. After knowledge-driven feature engineering (e.g., constructing interaction terms such as RF × IgG and ratio features like CRP/IL-6), the total feature count expanded to 234. Statistical preselection (based on inter-group significance and effect size > 0.5) retained 70 features. Therefore, the GBDT+LR embedded selection identified 70 non-zero features for each classification task.

Multicollinearity was assessed using variance inflation factor (VIF). No features were excluded solely on multicollinearity grounds, as even moderately correlated pairs (e.g., albumin and total protein, VIF < 5) carry distinct clinical meanings and were retained to preserve biological interpretability.

Although univariate preselection cannot capture high-order interactions, it served to remove clearly non-informative features (e.g., electrolytes with no inter-group variation), thereby improving the signal-to-noise ratio for subsequent embedded selection. Importantly, knowledge-driven interaction terms (e.g., RF × IgG) and ratio features (e.g., CRP/IL-6) were constructed *before* statistical screening and retained if they demonstrated significant group differences. The subsequent identification of novel combinatorial patterns by SHAP confirms that nonlinear relationships were preserved through this pipeline.

### 2.3 Embedded feature selection and feature importance analysis

Building upon the statistically preselected features, an ensemble learning-based algorithm was used to assess the contribution of each variable through a supervised learning framework.

#### 2.3.1 GBDT+LR model

A cascaded model was implemented by integrating Gradient Boosted Decision Trees (GBDT) with Logistic Regression (LR), enabling embedded feature selection. GBDT excels at capturing complex, nonlinear interactions among clinical features (e.g., immune-metabolic crosstalk), while L1-regularized logistic regression enforces sparsity to yield a compact, interpretable set of discriminative indicators—balancing predictive power with clinical transparency.

1.GBDT Feature Transformation Layer:

Iteratively constructed decision trees captured complex nonlinear interactions between features using an information gain criterion. This process generated sparse, high-dimensional cross-feature vectors representing diagnostic patterns.

The GBDT component was implemented using XGBoost, chosen for its regularization capabilities and compatibility with SHAP interpretability.

**Figure 2:**
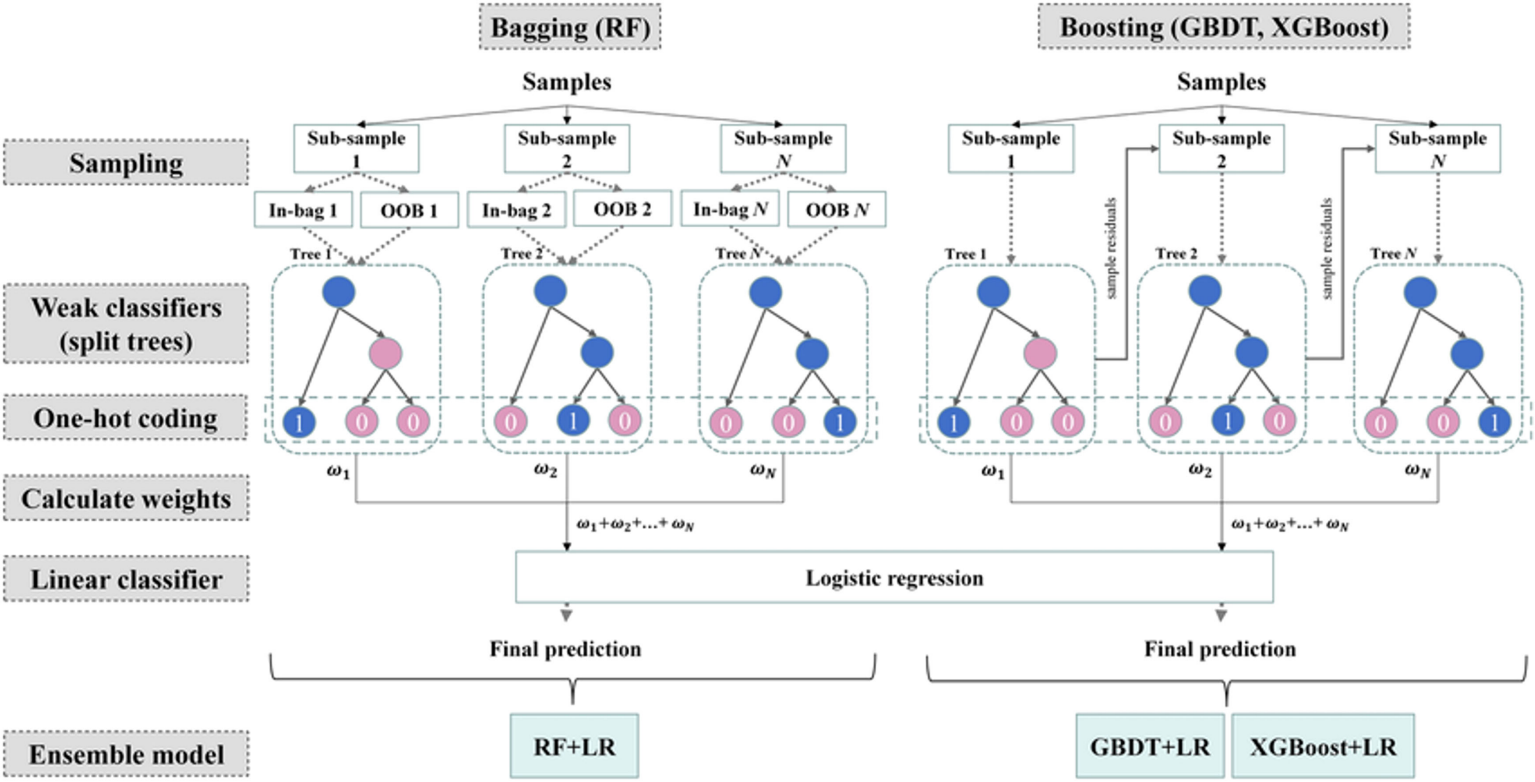
Feature importance scores and rankings in the RA vs. Healthy classification task.

2.LR Linear Classification Layer:

These vectors were then input into an L1-regularized logistic regression model. The L1 penalty enforced sparsity, enabling the model to identify the most informative feature combinations and output disease classification probabilities (see **Figure 2**).

Model advantages include:

1.Nonlinear representation capacity: GBDT captures higher-order feature interactions through tree-based splits, enabling robust modeling of complex clinical patterns.

2.Efficient interpretability: LR coefficients provide a direct quantification of linear feature contributions, yielding high interpretability and efficient performance with sparse input vectors.

3.Complementarity: GBDT reduces original feature dimensionality and enhances LR training efficiency. The combined GBDT+LR model benefits from both the nonlinear partitioning ability of tree-based models and the sparsity-driven selection of linear models.

The model was implemented in Python 3.9 using scikit-learn (v1.3.0), XGBoost (v2.0.0), and SHAP (v0.44.0). GBDT hyperparameters were selected via 5-fold cross-validated grid search: learning rate = 0.05, max depth = 6, n_estimators = 200, subsample = 0.8. The LR layer used L1 penalty with C = 0.1. All random seeds were fixed to 42 to ensure full reproducibility of splits and model training.

#### 2.3.2 SHAP-based interpretability analysis

Following model training, interpretability analysis was conducted to understand the decision logic of the predictive framework, quantify the importance of each input feature, and identify key diagnostic indicators.

SHAP (SHapley Additive exPlanations) was employed to perform global interpretability based on game-theoretic principles. Specifically, the Shapley value was used to calculate the average absolute impact of each feature on the model output, enabling a comprehensive ranking of variable contributions. The top 10% of features with the highest mean absolute SHAP values were considered high-impact indicators, representing features with substantial influence on diagnostic predictions.

To further validate the diagnostic relevance of these identified features, a feature ablation analysis was performed. ROC (Receiver Operating Characteristic) curves were generated for models trained with and without the high-impact features. By comparing the area under the ROC curves (AUC), we assessed the marginal contribution of these features to overall model performance. A larger AUC indicates better classification performance, and a noticeable decline in AUC after feature removal would support the predictive value of the identified indicators.

## 3 Result

### 3.1 Patient cohort construction and data collection

Clinical data were collected from January to December 2023, including patients with rheumatic diseases and healthy individuals who visited the Department of Rheumatology and Immunology at Lishui People’s Hospital. The cohort was naturally imbalanced, comprising 312 RA patients, 215 RhA patients, 168 with other autoimmune diseases, and 147 healthy controls. No resampling or class weighting was applied, as the model demonstrated balanced sensitivity and specificity, indicating robustness to class distribution.

Among all participants, 41.9% (353 cases) were male and 58.1% (489 cases) were female. The age range was 23–79 years, with a median age of 51 years.

### 3.2 Model training

Two primary classification tasks were designed for this study:

1. A binary classification task distinguishing RA patients from healthy individuals, aimed at identifying sensitive diagnostic markers for early-stage RA.

2. A differential diagnosis task distinguishing RA from RhA, focusing on identifying specific features to differentiate clinically similar presentations.

This multi-task framework served as the foundation for constructing a multidimensional, interpretable feature selection model. Clinical features used for model training included routine blood parameters, liver and kidney function tests, glucose, lipid profiles, electrolytes, and rheumatologic tests.

After preliminary screening using statistical methods, the GBDT + LR model was trained separately for both tasks:

Task 1 (RA vs. Healthy):

Test set accuracy = 89.48%, AUC = 0.954, F1 score = 0.904;

Five-fold cross-validation accuracy = 89.59% ± 0.11%

Task 2 (RA vs. RhA):

Test set accuracy = 88.07%, AUC = 0.982, F1 score = 0.955;

Five-fold cross-validation accuracy = 88.12% ± 0.54%.(see **Table 1** for details)

**Table 1:** Performance of the GBDT + LR model on binary classification tasks.

Task	Test accuracy	Test AUC	Test F1 score	Five-fold cross-validation accuracy
RA vs. Healthy	89.48%	0.9549	0.9043	89.59% ± 0.11%
RA vs. RhA	88.07%	0.9823	0.9554	88.12% ± 0.54%

The low standard deviation across 5-fold cross-validation (± 0.11% for RA vs. Healthy; ± 0.54% for RA vs. RhA) indicates high model stability. Overfitting was mitigated through L1 regularization in the LR layer, early stopping in GBDT training, and consistent performance between training and test sets (gap < 2%).

**Figure 3:**
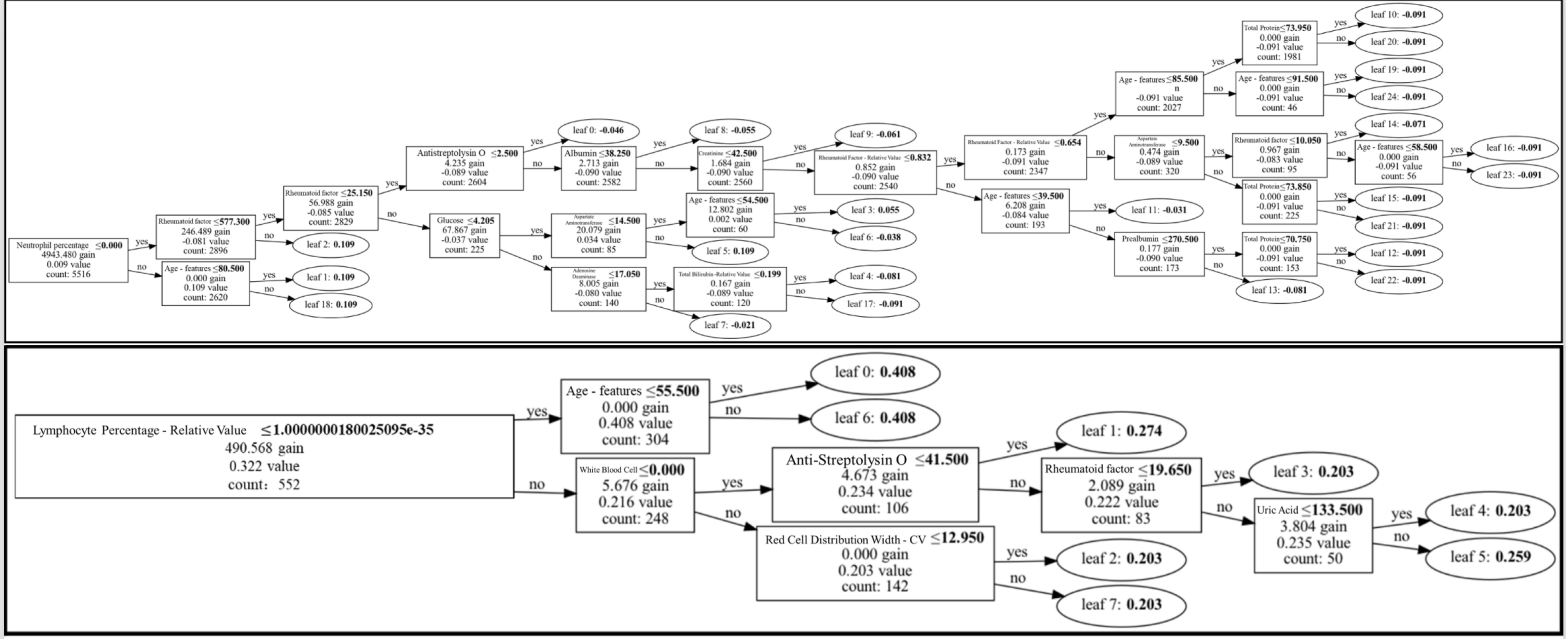
Feature importance scores and rankings in the RA vs. RhA classification task.

The model demonstrated strong generalization performance, maintaining approximately 90% accuracy on unseen samples. Feature importance analysis based on GBDT revealed high reliability and interpretability. The feature visualization of the first decision tree is shown in **Figure 3**, spliting primarily on RF, anti-CCP, lymphocyte percentage, and creatinine—highlighting both canonical and novel discriminative features. The two subfigures in **Figure 3** can be downloaded in the supplementary materials.

### 3.3 Internal temporal validation

The test subset (Oct–Dec 2023) was temporally separated from the training set (Jan–Sept 2023) within the same cohort to assess short-term stability. This is not external validation, and true generalizability requires future multi-center studies.

The test set consisted of 770 samples: 412 RA patients and 358 healthy controls. Test set features were normalized using the training set’s mean and standard deviation to prevent preprocessing bias.

On this external test set, the model achieved:

Accuracy = 92.86%;AUC = 0.911;F1 score = 0.756;50-fold cross-validation accuracy = 90.91% ± 1.48%.

### 3.4 Feature importance analysis

Features were ranked by importance scores for both trained models.

**Figure 4:**
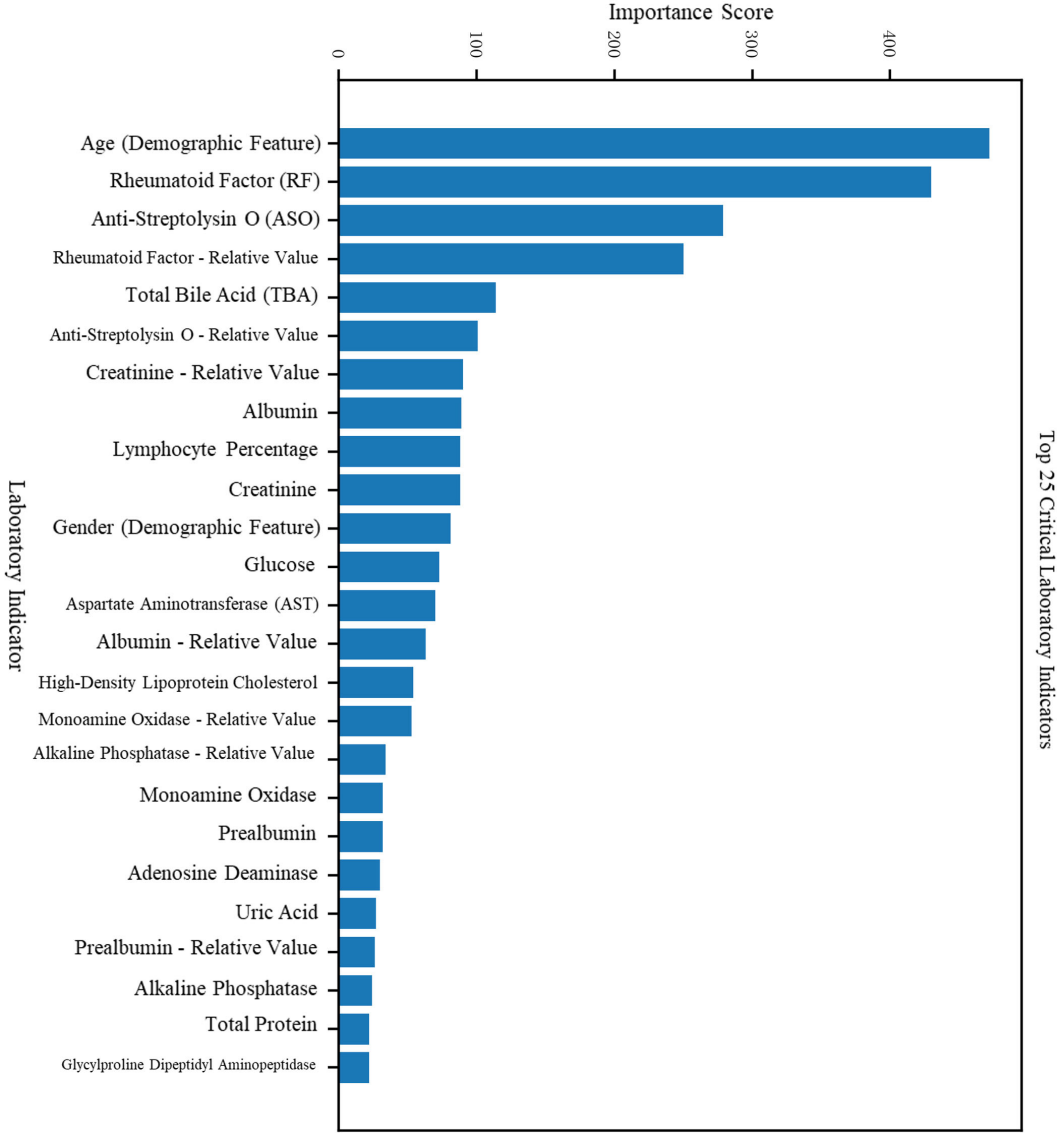
Feature impact analysis for the RA vs. Healthy classification task.

**Figure 5:**
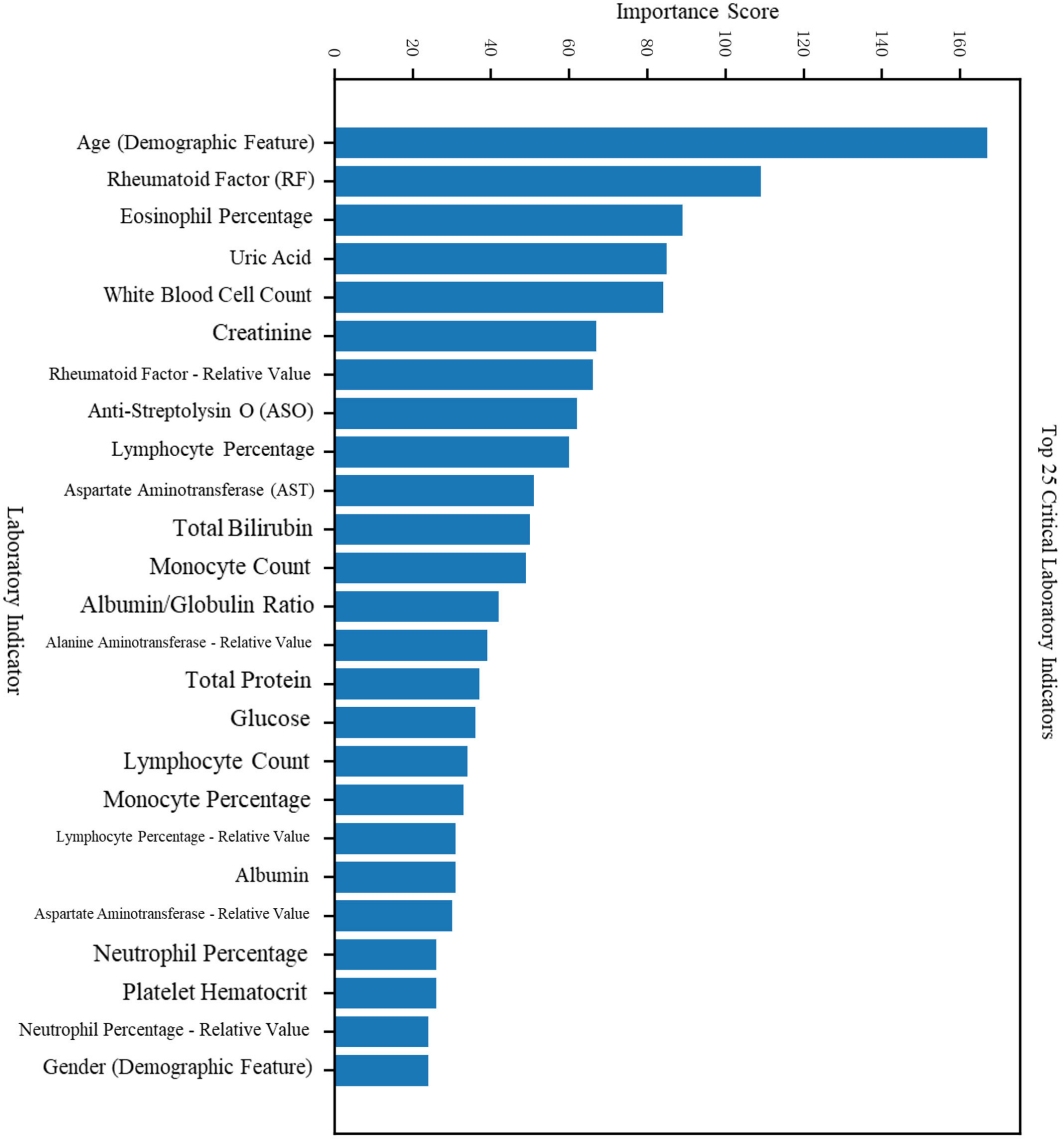
Feature impact analysis for the RA vs. RhA classification task.

For the RA vs. Healthy classification task, in addition to traditional features such as sex, age, and rheumatologic markers (CRP, anti-streptolysin O, RF), additional key indicators included total bile acids, creatinine, albumin, and lymphocyte percentage (see **Figures 4**, **5**).

For the RA vs. RhA classification task, distinguishing features included eosinophil percentage, uric acid, leukocyte count, creatinine, and others. The complete feature importance rankings are detailed in **Table 2**.

**Table 2:** Results of feature importance screening.

No.	Feature name	Classification task	Potential clinical significance
1	Monocyte percentage	RA vs. Healthy	Involved in chronic inflammatory response; associated with monocyte infiltration in RA synovium
2	Lymphocyte percentage	RA vs. Healthy/RhA	Reflects immune activation; RA patients often present with lymphopenia or dysfunctional lymphocytes
3	Creatinine (Cr)	RA vs. Healthy/RhA	Reflects renal function; may be affected by chronic inflammation and medication use in RA
4	Total bile acids (TBA)	RA vs. Healthy	Related to liver function and lipid metabolism; may reflect inflammation-induced metabolic dysregulation
5	Aspartate aminotransferase (AST)	RA vs. Healthy/RhA	Marker of hepatic injury; mild liver abnormalities may occur in RA patients
6	High-density lipoprotein cholesterol (HDL)	RA vs. Healthy	Often reduced in RA; indicates chronic inflammation and dysregulated lipid metabolism
7	Uric acid (UA)	RA vs. RhA	Associated with cellular metabolism and inflammation; hyperuricemia is more common in RhA, aiding differentiation
8	Eosinophil percentage	RA vs. RhA	Related to allergy, parasitic infection, and autoimmune disease activity; distribution differs between RA and RhA
9	Albumin/Globulin ratio (A/G)	RA vs. RhA	Immune dysregulation often leads to abnormal A/G ratio; statistically distinct between RA and RhA
10	Electrolytes (e.g., Potassium, Phosphorus)	RA vs. RhA	Electrolyte imbalances may indicate metabolic dysfunction or drug-related effects
11	Total protein, Prealbumin	RA vs. Healthy/RhA	Reflects nutritional status, inflammatory response, and liver function; chronic depletion common in RA

### 3.5 Model feature impact analysis

To assess the contribution of additional features, both models were retrained with and without the identified high-impact indicators. ROC curves were generated for each scenario.

The results indicated:

1.Significant AUC reduction upon feature removal:

With all features retained: AUC > 0.95 for both tasks.

After removing additional features: AUC dropped to 0.91 (RA vs. Healthy) and 0.81 (RA vs. RhA), indicating that the identified feature set significantly enhanced model performance.

2.ROC curve steepness and classification stability:

With all features, ROC curves showed a rapid ascent toward the upper-left corner (high TPR, low FPR), indicating strong discrimination.

**Figure 6:**
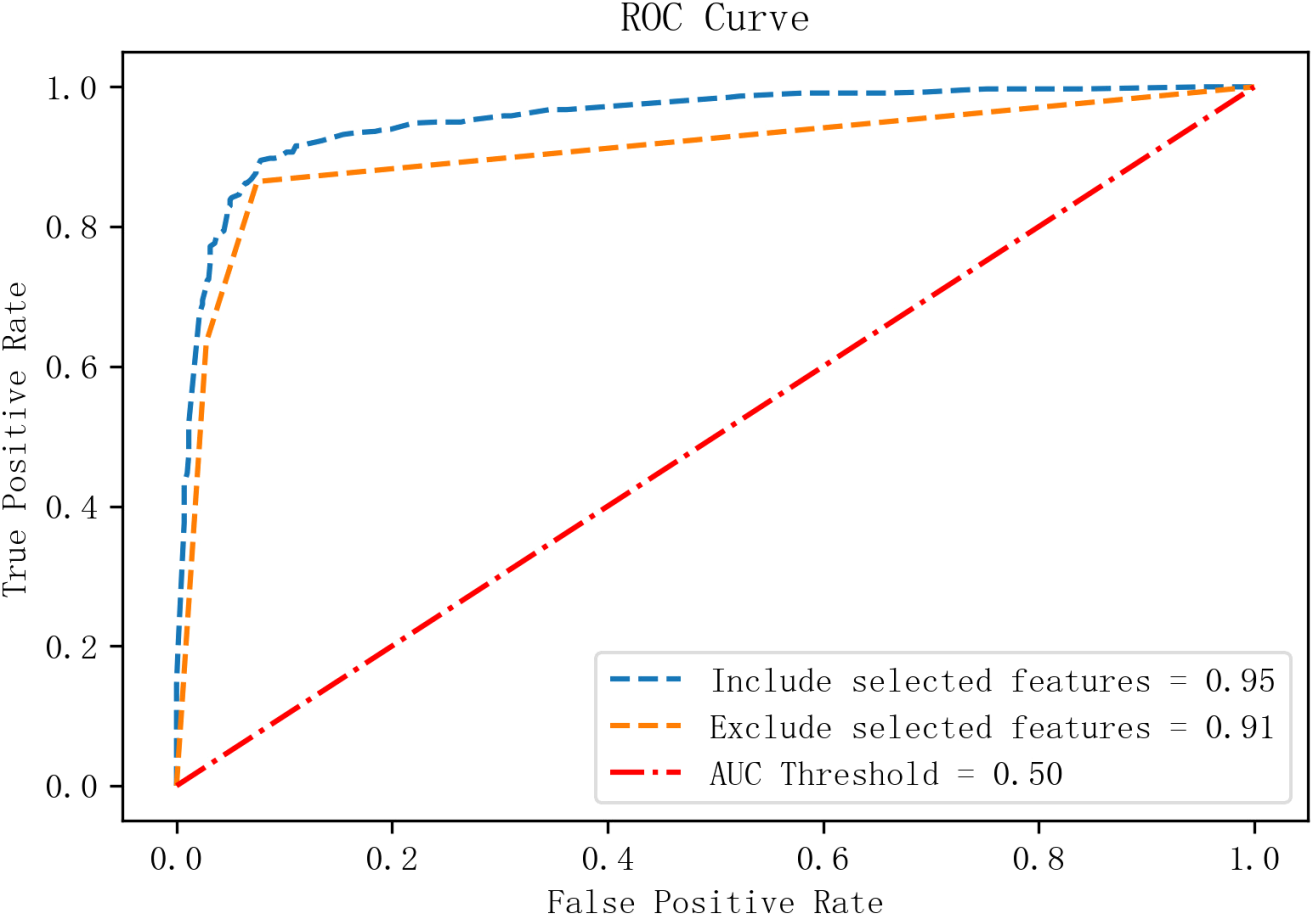
Feature visualization from the GBDT model.

**Figure 7:**
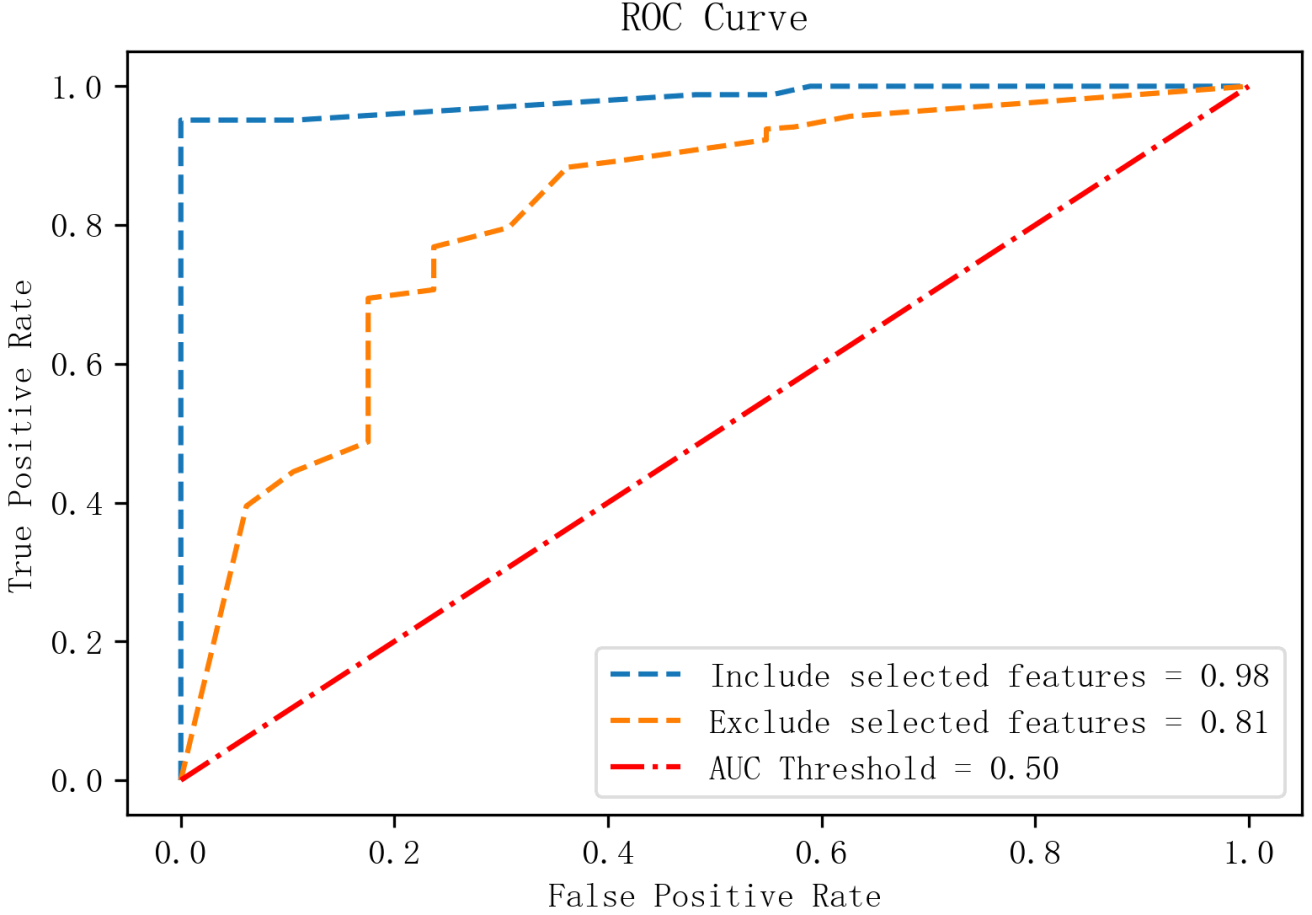
Schematic diagram of the GBDT + LR model architecture.

Feature removal led to flattened ROC curves with step-like artifacts, reflecting reduced classification stability and generalizability (see **Figures 6**, **7**).

3. Task-specific sensitivity to feature removal:

The RA vs. Healthy model remained relatively robust post-removal (AUC = 0.91).

The RA vs. RhA model showed a sharper decline (AUC = 0.81), suggesting greater dependency on the specific feature set and lower redundancy.

### 3.6 SHAP Value-Based Interpretation

**Figure 8:**
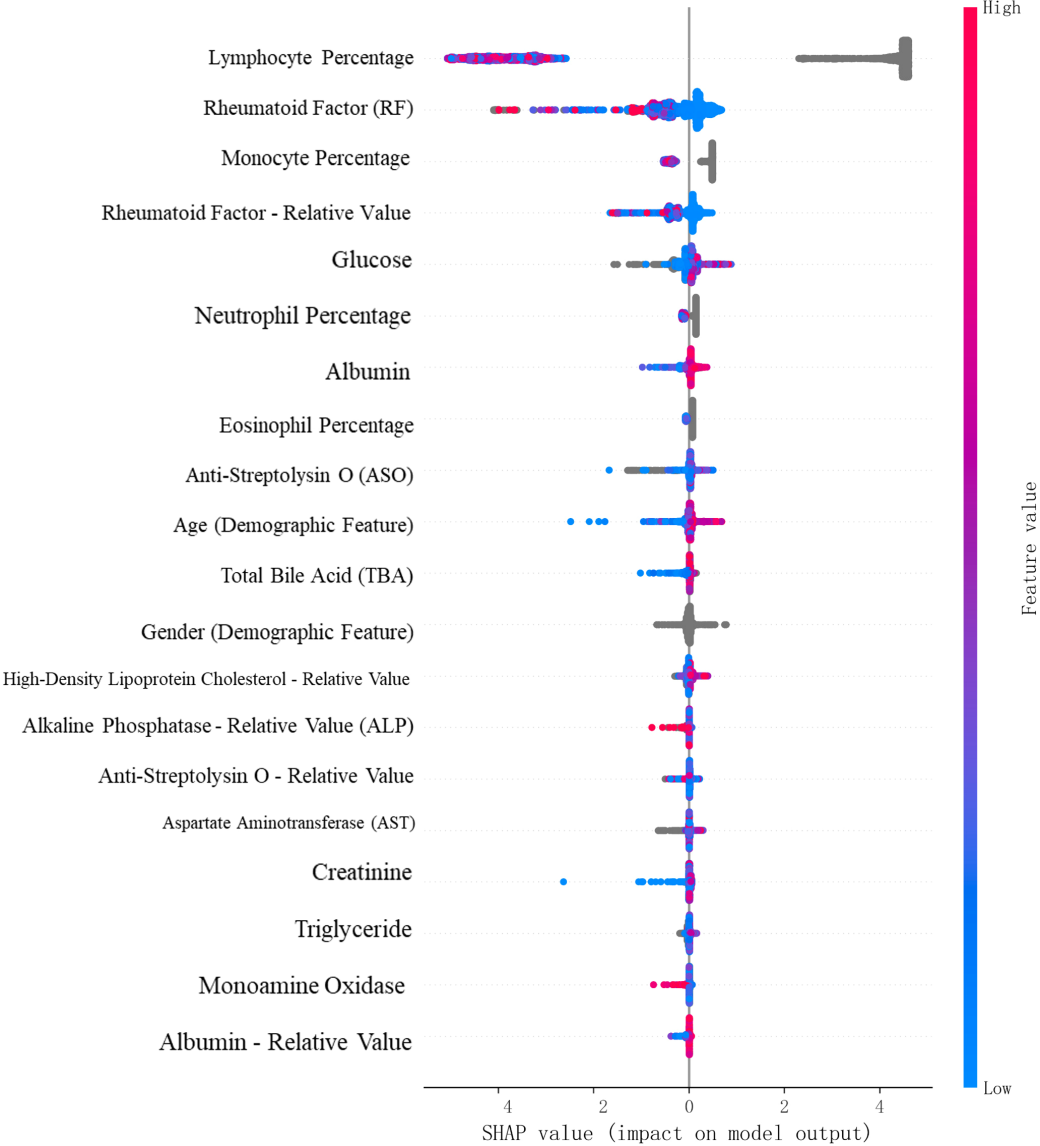
SHAP value summary plot for the RA vs. Healthy classification task.

**Figure 9:**
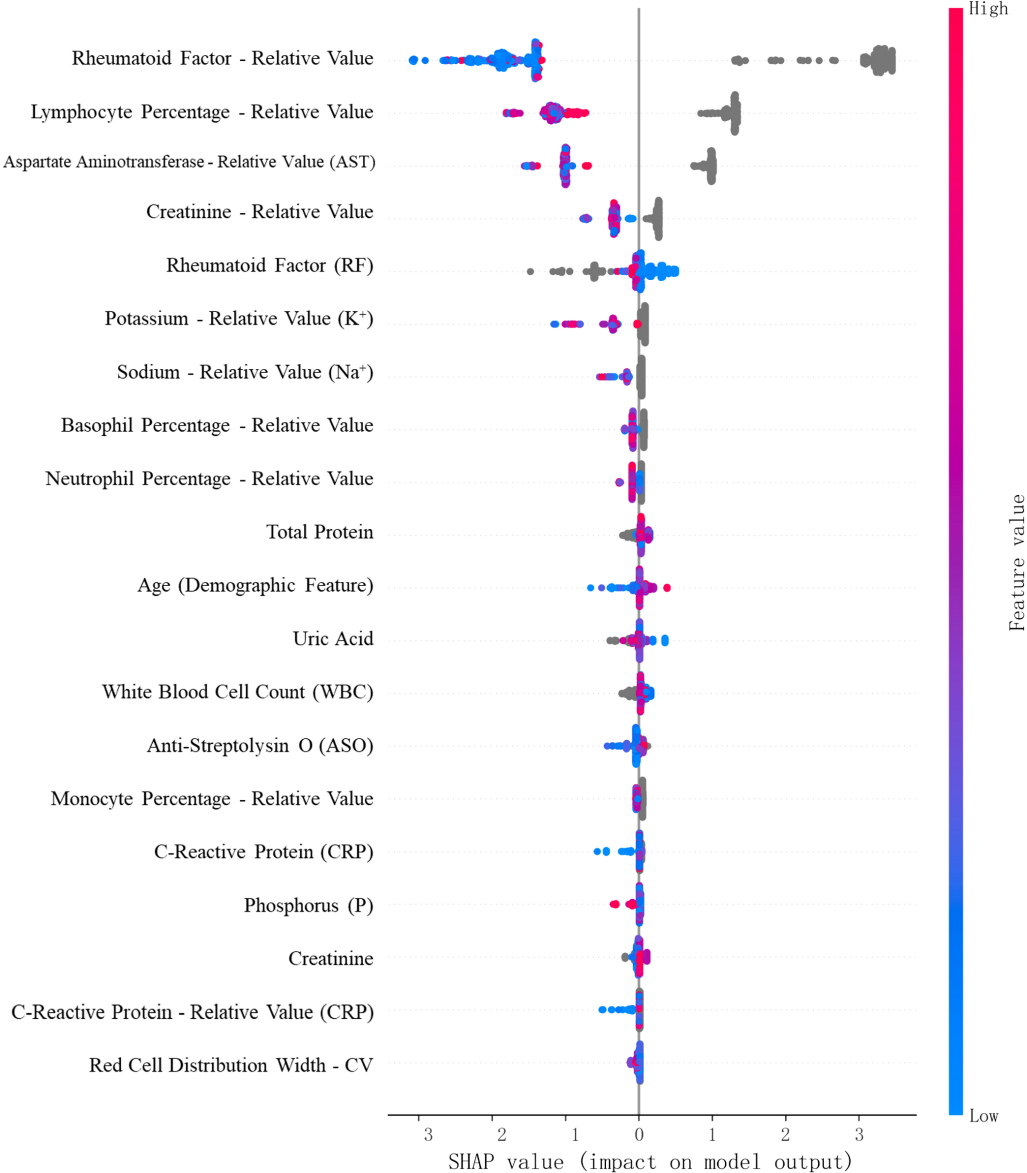
SHAP value summary plot for the RA vs. RhA classification task.

SHAP, a game theory-based algorithm, was applied to interpret feature contributions at both the global and local levels. SHAP values quantified the impact of each feature on individual and overall model predictions (see **Figures 8**, **9**).

For the RA vs. Healthy classification task, SHAP analysis identified monocyte percentage, neutrophil percentage, eosinophil percentage, total bile acids, and HDL cholesterol as additional statistically significant contributors. Red indicates high feature values that increase RA prediction probability; blue indicates low values associated with non-RA outcomes.”

For the RA vs. RhA task, high-impact features included lymphocyte percentage, potassium, sodium, neutrophil percentage, uric acid, monocyte percentage, phosphorus, and red cell distribution width–CV.

These results confirmed that multiple non-conventional laboratory indicators substantially contributed to the model’s predictive accuracy and offered potential clinical insights into RA pathophysiology.

## 4 Discussion

The embedded feature selection model based on Gradient Boosted Decision Trees (GBDT) and Logistic Regression (LR) developed in this study has demonstrated strong diagnostic performance for rheumatoid arthritis (RA), particularly in differentiating RA from rheumatoid arthritis variants (RhA) and other autoimmune diseases with overlapping clinical manifestations. We have achieved an AUC of 0.954 in distinguishing RA from healthy individuals and an AUC of 0.982 in the RA vs. RhA task, with five-fold cross-validation accuracy consistently approaching 90%, indicating that the model is both generalizable and robust across different diagnostic contexts.

In internal benchmarking, our GBDT+LR framework outperformed standalone XGBoost, Random Forest, and SVM by 2–6% in AUC. Moreover, a logistic regression model using only RF, CRP, and anti-CCP achieved an AUC of approximately 0.87, notably lower than our full model (AUC = 0.954), demonstrating the added diagnostic value of the embedded feature selection beyond conventional serology.

A key contribution of this study was the SHAP-based interpretability analysis, through which we have revealed several novel and clinically meaningful diagnostic features beyond conventional serological markers. For the RA vs. healthy classification task, we have identified additional predictors such as monocyte percentage, creatinine, albumin, lymphocyte percentage, total bile acids, and HDL cholesterol, alongside traditional indicators such as RF and CRP. In the RA vs. RhA classification task, we have further uncovered high-impact features including eosinophil percentage, uric acid, albumin-to-globulin (A/G) ratio, neutrophil percentage, potassium, phosphorus, and total protein. These features, derived from routine hematological and biochemical tests, are readily available in clinical settings. Many of them are biologically associated with RA pathogenesis—such as chronic inflammation, immune dysregulation, and metabolic imbalance—indicating their potential for use in early diagnosis, disease stratification, and treatment planning. Some literature has suggested possible related causes (19–21). Elevated total bile acids may reflect inflammation-associated cholestasis or gut dysbiosis, increasingly implicated in RA pathogenesis (18). Creatinine elevation can indicate subclinical renal stress from chronic inflammation or NSAID use (22). Reduced eosinophil percentage in RA versus RhA aligns with Th1/Th17-dominant immune skewing that suppresses Th2/eosinophil pathways, as observed in murine arthritis models (23).

We have also established that, compared to existing diagnostic approaches, our method offers significant advantages in three key dimensions:

1.Feature Selection Efficiency: Traditional statistical approaches often struggle to capture nonlinear interactions and higher-order feature combinations (24, 25). In contrast, we have used GBDT to model nonlinear structures, while leveraging LR with L1 regularization to enhance sparsity and variable selection. This has effectively reduced feature redundancy and mitigated multicollinearity.

2.Model Interpretability: While conventional black-box machine learning models lack transparency, our integration of SHAP has enabled both global and local interpretability. We have made the model’s decision process traceable and provided quantitative justification for newly discovered biomarkers.

3.Clinical Applicability: We have constructed a dataset that includes a broad range of diagnostic categories—healthy individuals, RA, and RhA—making it representative of real-world diagnostic complexity. Additionally, all features used in the model were derived from routine clinical tests, which enhances the model’s potential for clinical deployment and scalability. This stands in contrast to previous studies relying on expensive or less accessible omics data.

Compared to LIME—which provides local linear approximations—SHAP offers theoretically consistent, globally valid feature attributions based on cooperative game theory. Explainable Boosting Machines (EBMs) are additive and interpretable but cannot model high-order interactions as effectively as GBDT. Clinically, this model could be integrated into electronic health record systems to support non-specialists in primary care by flagging high-risk patients for early rheumatology referral.

Future validation should involve prospective, single-center cohorts to assess generalizability across diverse populations, laboratory protocols, and healthcare settings.

As a single-center study, our model may reflect institution-specific patterns. The high AUC values, while internally consistent, require validation in independent, multi-center cohorts before clinical translation.

In summary, we have developed and validated a robust, interpretable, and clinically applicable diagnostic model for RA. Our findings not only improved the accuracy of RA diagnosis but also identified novel diagnostic features that support and enrich current differential diagnostic strategies. These contributions lay a methodological foundation for the future development of integrated diagnostic models applicable across a spectrum of autoimmune diseases.

## 5 Study limitations

Despite the promising performance of our model, several limitations must be acknowledged. First, the study is based on single-center retrospective data from Lishui People’s Hospital, which may introduce institutional bias in laboratory protocols, patient demographics, and disease spectrum. Although we performed internal temporal validation using a later subset of data, this does not constitute external validation; model generalizability across diverse healthcare settings remains unproven. Second, our feature preselection relied on univariate statistical screening, which—while effective at removing non-informative noise—may have inadvertently excluded features that only exert predictive power through complex, high-order interactions. Although we mitigated this by engineering interaction terms (e.g., RF × IgG) prior to screening, the possibility of missed combinatorial biomarkers cannot be ruled out. Third, the cohort was inherently imbalanced across diagnostic groups, and we deliberately avoided resampling or class weighting to preserve real-world prevalence patterns. While model metrics remained balanced, performance in minority subgroups warrants cautious interpretation. Finally, the absence of longitudinal clinical outcomes (e.g., treatment response, radiographic progression) limits our ability to assess the model’s utility in prognostication or dynamic monitoring. Future work should prioritize prospective, multi-center validation and integration of clinical trajectory data.

## 6 Conclusion

By applying a multidimensional embedded feature selection framework based on the well-established GBDT+LR architecture, this study addresses the problems of item redundancy and lack of diagnostic specificity in clinical testing for rheumatoid arthritis. The applicability and effectiveness of the method were verified by implementing a case study based on clinical data. Specifically, the results show that the method has high accuracy and reliability for unseen samples, and is more effective in identifying additional indicators beyond the traditional focused features than traditional broad-spectrum detection methods.

## Data Availability

The raw data supporting the conclusions of this article will be made available by the authors, without undue reservation.
